# Physiological Perturbations in Combat Sports: Weight Cycling and Metabolic Function—A Narrative Review

**DOI:** 10.3390/metabo14020083

**Published:** 2024-01-24

**Authors:** Modesto A. Lebron, Jeffrey R. Stout, David H. Fukuda

**Affiliations:** Physiology of Work and Exercise Response Laboratory, Institute of Exercise Physiology and Rehabilitation Science, University of Central Florida, Orlando, FL 32816, USA; modesto.lebron@ucf.edu (M.A.L.); jeffrey.stout@ucf.edu (J.R.S.)

**Keywords:** combat sports, weight cycling, metabolic flexibility, low energy availability

## Abstract

Combat sports athletes seeking a competitive edge often engage in weight management practices to become larger than their opponents, which ultimately includes periods of gradual weight loss, rapid weight loss, and weight regain. This pattern of weight loss and regain is known as weight cycling and often includes periods of low energy availability, making combat sports athletes susceptible to metabolic dysfunction. This narrative review represents an effort to explore the metabolic perturbations associated with weight cycling and outline the short-, medium-, and long-term effects on metabolic flexibility, function, and health. The short-term effects of rapid weight loss, such as a reduced metabolic rate and alterations to insulin and leptin levels, may prelude the more pronounced metabolic disturbances that occur during weight regain, such as insulin resistance. Although definitive support is not currently available, this cycle of weight loss and regain and associated metabolic changes may contribute to metabolic syndrome or other metabolic dysfunctions over time.

## 1. Introduction

Combat sports encompass activities that involve striking (e.g., boxing, muay thai), grappling (e.g., jiu jitsu, judo, wrestling), or both (e.g., mixed martial arts). Athletes in these sports often compete in weight-specific categories, aiming to minimize significant differences in body mass among competitors [1]. Despite this, it is common for athletes to engage in weight management practices to become larger than their opponents, seeking a competitive edge [2]. A prevalent method is rapid weight loss (RWL), involving fluid and dietary restrictions, heat exposure, and increased physical activity, typically executed shortly before a competition. This is frequently followed by a period of time with rapid weight regain [3]. Research, such as that by Lakicevic et al. [4], has documented athletes reducing their body mass by 2% to over 10% before a competition, only to regain or exceed their initial weight in the subsequent days or weeks. This pattern of weight loss and regain, known as weight cycling, is prevalent and varies across different combat sports. For instance, Artioli et al. [5] found that active judo athletes could undergo up to 10 or more cycles of weight reduction annually.

The impact of rapid weight loss on physical performance in combat sports is a significant focus of research, exploring areas like aerobic, anaerobic, and cognitive function [6]. Moreover, extensive research and reviews have discussed the ethical concerns regarding RWL in combat athletes [7]. However, the relationship between RWL and competitive success is not well established. Brechney et al. [8] studied 75 mixed martial artists (59 amateurs, 16 professionals) and found no significant difference in the percentage of body mass lost between winners (6.8%) and losers (7.4%) of fights. Interestingly, they discovered an inverse relationship between the amount of body mass lost and the likelihood of winning, with each unit of body mass lost correlated with an 11% decrease in the odds of reporting a win, with an odds ratio of 0.89 (CI: 0.79–1.00). In contrast, Faro et al. [9] observed a positive correlation in a larger study of 700 mixed martial artists: rapid weight gain after weigh-ins was associated with a higher probability of winning, with each 1% increase in body weight correlating with a 4.5% increase in the chance of winning. These findings suggest that rehydration following weigh-ins, which is believed to mitigate the adverse effects on anaerobic performance [10], could play a role in enhancing performance. Similar benefits have been noted for aerobic performance in active and endurance-trained individuals [11].

Overall, the relationship between weight fluctuation and competitive success in combat sports remains ambiguous. However, the repetitive nature of weight cycling might lead to physiological changes that increase the risk of metabolic dysfunction during an athlete’s career and potentially after retirement. This narrative review aims to explore metabolic perturbations within this population, addressing concerns about metabolic flexibility and dysfunction due to low energy availability (LEA), RWL, and weight cycling.

## 2. Metabolic Health

Weight cycling in combat sports athletes may also include periods of LEA, traditionally defined as <30 kcal·kg·FFM·day^−1^ [12]. LEA has been observed to have a wide variety of impacts on metabolic function, such as reductions in insulin, leptin, and thyroid hormones (free and total T3), which regulate substrate utilization [13] and/or metabolic rate [14] and may ultimately influence metabolic flexibility. Metabolic flexibility can be described as the ability of skeletal muscle to maintain energy homeostasis by adjusting the utilization of substrate pathways [13,15]. The efficiency of metabolic adjustments to available energy substrates is critical during transitions from post-absorptive (fasted) to absorptive (fed) states and from rest to exercise [15,16]. As the final recipients of metabolic substrates, mitochondria and their ability to adjust substrate utilization in response to changes in nutritional and/or physical activity are critical to metabolic flexibility [13]. As such, metabolic adjustments lacking ease and rapidity have been termed metabolic inflexibility and are associated with a variety of negative health implications, including metabolic syndrome. Metabolic syndrome is characterized by several metabolic risk factors related to abdominal obesity, impaired glucose tolerance, dyslipidemia, and high blood pressure that may ultimately increase the risk of cardiovascular disease or the development of diabetes mellitus [17]. This may be of concern for combat sports athletes, as previous research has identified an increased risk of post-career obesity in weight category athletes when compared to non-weight category athletes [18].

The purpose of this review is not an exhaustive description of the precise cellular mechanisms that regulate metabolic flexibility, as comprehensive reviews of regulation and dysfunction can be found elsewhere [13,19]. However, there are several key factors relevant to combat sports athletes and weight cycling. For example, metabolic flexibility is highly dependent on endocrine hormones such as insulin and leptin, where insulin regulates factors such as fatty acid and glucose oxidation, and leptin serves as an indicator of the available stored energy within the body [13]. In addition, previous work has regarded lactate as a regulator of substrate metabolism describes fasting plasma lactate to reflect mitochondrial substrate utilization, thereby a proxy for metabolic flexibility [20].

Generally, long-term exercise is beneficial in mitigating the physiological abnormalities related to metabolic inflexibility that are associated with metabolic syndrome [13]. In a study of former athletes and non-athletes, Batista and Soares [21] identified former athletes to have a lower prevalence of metabolic syndrome. It is important to note that confounding factors revealed that only former athletes who then met the recommended levels of weekly physical activity were less likely to be affected. Moreover, weight cycling, as demonstrated by weight category athletes in combat sports, may ultimately result in physiological perturbations in body composition and metabolism, leading to risk factors associated with a decreased in health status, regardless of regular exercise. This may include, but is not limited to, increased weight gain after weight loss, hyperinsulinemia, and dyslipidemia [22].

Additionally, although a large proportion of research related to RWL in combat sports is conducted in males, female combat athletes have been observed to partake in similar weight loss strategies prior to competition [23,24,25]. Female combat sports athletes demonstrated no difference in rapid weight gain when compared to males (8.0% ± 3.8% vs. 8.1% ± 3.6%, respectively). Although the scope of this manuscript does not permit an exhaustive description of all physiological dysregulations involved, extended periods of LEA may increase the risk of the female athlete triad, or relative energy deficiency in sport, with females potentially more sensitive to reductions in energy availability [26]. Previous work has observed a significant (*p* = 0.013, r = 0.244) relationship between weight cutting habits and the female athlete triad, with 38% of the sampled female combat athletes at risk of developing the triad [27]. Taken together, with similar weight loss and regain strategies employed by females compared to males and greater vulnerability to physiological dysregulation, weight cycling in female combat athletes may require additional considerations.

As primary research on acute alterations in metabolic flexibility and LEA in combat sports athletes and their long-term effects on metabolic dysfunction is lacking, additional information from various populations may be used to identify short-, medium-, and long-term effects. For example, Melin and colleagues [28] utilized this approach to identify potential direct and indirect influences of LEA on performance in active and athletic populations, such as endurance athletes, combat sports athletes, and bodybuilders. As such, the following section describes observations related to metabolic function, LEA, RWL, and weight cycling.

## 3. Metabolic Perturbations

### 3.1. Short Term

For combat sports athletes, a short-term perturbation can be defined as an event that occurs over days to weeks and is generally equivalent to a single cycle of RWL. At the professional level, flyweight (125 lb.) mixed martial artists have been observed to lose approximately 9–11 pounds during the 72-h period before weighing in for competition and demonstrate a weight regain of approximately 12–17 pounds in the 24 h following [29]. Similarly, a survey of competitive judokas over the age of 12 years old (19.3 ± 5.3 years) showed that 82% were regularly engaged in weight loss practices, at an average weight reduction of 5% body weight [5]. Moreover, 34% of jiu jitsu athletes have been observed to reduce their body weight by 2.3 ± 4.6% kg between 0 and 7 days prior to a competition [30]. This magnitude of RWL may be accompanied by a variety of metabolic perturbations, as a result of weight reduction, dehydration, energy restriction, thermal stress, and/or a combination of multiple factors, including decreases in resting metabolic rate, insulin, and leptin [31,32,33].

#### 3.1.1. Combat Athletes

In a study conducted by Sagayama and colleagues [32], the metabolic rate was observed in weight-classified boxers and judokas before and after self-prescribed RWL (−6.0% ± 0.9%) over one week. To achieve weight loss, participants demonstrated dietary restrictions (mean intake at baseline: 2458 ± 578 kcal·day^−1^ vs. weight loss: 1008 ± 354 kcal·day^−1^), self-reported decreased fluid intake, and increased physical activity (energy expenditure at baseline: 1.88 ± 0.13 kcal·min^−1^ vs. weight loss: 1.93 ± 0.14 kcal·min^−1^). The sleeping metabolic rate significantly decreased from baseline (1822 ± 191 kcal·day^−1^) to weight loss (1587 ± 150 kcal·day^−1^), while a nonsignificant (*p* = 0.07) trend was noted for the basal metabolic rate (1998 ± 242 vs. 1846 ± 161 kcal·day^−1^). Similar findings were observed in an elite taekwondo athlete, where a 5-day period of LEA (<10 kcal·kg·FFM·day^−1^) subsequently resulted in a reduction in the measured resting metabolic rate (−149 kcal·day^−1^) and the ratio of measured versus predicted resting metabolic rate (0.94–0.87) [34]. However, the authors noted that dietary restrictions and associated diet-induced thermogenesis may have caused a decrease in sleeping metabolic rate. In a study with lean and obese individuals undergoing a 48-h period of fasting, eucaloric feeding, or overfeeding, the sleeping metabolic rate decreased significantly (4.4 ± 7.76%) in response to the fasting [35]. 

With previous investigations of substrate utilization and metabolic flexibility conducted primarily in non-combat sports athletes, it is critical to explore indicators of metabolic flexibility in combat sports athletes. In a study conducted by Artioli and colleagues [10], fourteen male judo competitors were separated into two groups (weight-loss = 7, control = 7) and completed two testing batteries consisting of judo-specific activity and upper-body Wingate assessments. The two testing batteries were separated by 5–7 days, during which the weight loss group was required to lose approximately 5% of their body weight using their typical strategies used before a competition, which ultimately consisted of energy and fluid restrictions, exercise, and/or heated environments. After attaining the desired body weight, the weight loss group was allotted a 4-h recovery period, where participants were able to refeed and rehydrate ad libitum (1391 ± 375 kcal energy intake; 201 ± 62 g carbohydrates; 50 ± 16 g fat; 34 ± 23 g protein). Plasma lactate was measured before and after the testing battery, as well as twice during the battery. No differences were observed in plasma lactate when comparing the weight loss and control groups at any time point, suggesting a minimal influence of RWL on mitochondrial function when a recovery period is allotted. Mendes and colleagues [36] observed similar findings in combat athletes with a grappling background (judo, jiu jitsu, wrestling, mixed martial arts) losing 5% of their body weight. Participants decreased their body mass for no more than 5 days using their typical strategies used before a competition and were given a recovery period of 4 h to eat and drink ad libitum. Participants completed a high-intensity intermittent exercise assessment before and after reducing their body mass, and plasma lactate was collected at baseline and after the assessment. Ultimately, resting plasma lactate was not affected by RWL when utilizing a 4-h recovery period to refeed and rehydrate. Moreover, no differences were observed in post-exercise plasma lactate measurements between assessments.

Degoutte and colleagues [31] observed that RWL of 5% body mass over 7 days in national-level judo competitors, primarily in the form of dietary and fluid restriction, resulted in a significant decrease in insulin by approximately 37%. During this period, participants in the weight loss group were reported to have consumed 7.0 ± 1.2 MJ·day^−1^, or approximately ~1673 kcal·day^−1^. When considering the mean value of fat-free mass (64.5 ± 1.1 kg), participants in this study would have been at risk of LEA (~26 kcal·kg^−1^·FFM·day^−1^) [8]. In contrast, in a study conducted by Talaee and colleagues [33], a group of wrestlers decreased their body mass by 4% over 1 week through the use of caloric (1700 ± 100 kcal·day^−1^) and water restriction (110 ± 95 mL·day^−1^), but were prohibited from the use of saunas, rubber clothes, intense exercise, diuretics, or laxatives. Following weight reduction, the athletes did not demonstrate a significant decrease in insulin but did demonstrate a significant decrease in leptin. However, the blood sample was collected 12 h after the weigh-in, making it unclear whether or not the athletes had dietary restrictions during this window. Similar findings have been observed in elite wrestlers, with a 19-day period of caloric restriction (~28 kcal·kg^−1^·day^−1^) demonstrating no impact to resting lactate or insulin levels [37]. It is important to note that LEA is typically in reference to fat-free mass; therefore, the caloric restriction employed by Mourier and colleagues [37] may not have been large enough to induce the metabolic changes associated with LEA.

#### 3.1.2. Non-Combat Athlete Comparisons

Beyond the observed effects on metabolic rate, substrate utilization may be affected by periods of RWL. In a situation similar to that experienced by combat sports athletes during RWL, Koehler et al. [38] evaluated aerobically trained healthy males during a 4-day period of LEA (15 kcal·kg·FFM·day^−1^) through means of dietary restriction and exercise. Body mass losses of approximately 2 kg and a substantial decrease in carbohydrate consumption (LEA: 1.6 ± 0.2 g·kg^−1^·day^−1^, LEA and exercise: 3.1 ± 0.3 g·kg^−1^·day^−1^, control: 4.0 ± 0.2 g·kg^−1^·day^−1^, control and exercise: 5.6 ± 0.5 g·kg^−1^·day^−1^) were accompanied by a decrease in serum glucose and an increase in free fatty acids. This may be indicative of sufficient substrate utilization adjustments even during acute periods of LEA in trained healthy individuals. Ultimately, healthy aerobically trained individuals may have the ability to undergo short periods of LEA without dramatic consequences to substrate utilization acutely, and they may efficiently adjust their utilization to the available source of energy. Combat sports athletes may demonstrate aerobic capacities that are similar to those of aerobically trained individuals. The individuals in this study [38] demonstrated a VO_2peak_ value of 49.3 ± 2.4 mL·kg^−1^·min^−1^, whereas previous research observing various combat sports athletes observed VO_2max_ values ranging from approximately 55 to 59 mL·kg^−1^·min^−1^ [39]. As such, combat sports athletes may also demonstrate similar capabilities related to undergoing periods of LEA and demonstrating sufficient adjustments to substrate utilization. However, additional considerations must be made regarding the nature of intermittent intensities in combat sports and their potential influences on substrate utilization adjustments. For example, during matches, Greco-Roman wrestlers and jiu jitsu competitors have been observed to demonstrate a work:rest ratio of approximately 2.5:1 and 6:1, respectively [40,41]. However, to the best of our knowledge, the effects of the training intensity, intermittency, duration, or competitive level between different combat sports on substrate utilization are unclear.

As described earlier, fasting plasma lactate may serve as a proxy for substrate utilization and metabolic flexibility [20]. In support, San-Millán and colleagues [42] identified that blood lactate was negatively correlated with fat oxidation (r = −0.92–0.98, *p* < 0.01) and positively correlated with carbohydrate oxidation (r = 0.94–0.99, *p* < 0.01) in international male professional cyclists, moderately active males, and males with metabolic syndrome during graded leg cycling to volitional exhaustion. Moreover, correlations between lactate and fat oxidation (r = −0.92–0.98) were observed; however, the groups differed in their capacity to oxidize fat, implying differences in mitochondrial function. Taken together, the reduction in lactate thresholds during exercise may indicate decreased mitochondrial function.

While there is little additional research exploring the effects of hypohydration or RWL on lactate parameters in combat sports athletes, research has been conducted in healthy and athletic populations that mimic the RWL tendencies of combat sports athletes. In a study conducted by Papadopoulos et al. [43], a group of healthy participants completed four randomly assigned VO_2max_ tests in which they were euhydrated (EU) or hypohydrated (HH) and in a thermal neutral environment (RM) or a hot/humid environment (HT). Before the hypohydration testing, participants were required to decrease their body mass by 2–4% by exercising for 30 min on a treadmill at 50% of their VO_2max_. Ultimately, resting lactate did not differ significantly in any condition (EU-RM = 1.74 ± 0.21 mmol, HH-RM = 1.47 ± 0.12 mmol, EU-HT = 1.50 ± 0.16 mmol, HH-HT = 1.51 ± 0.15 mmol) and the hydration status did not affect the lactate threshold. However, during a study conducted by England et al. [44], recreational cyclists completed two incremental cycle ergometer tests in either a euhydrated or hypohydrated state, consisting of approximately 5% body weight lost via sauna exposure. When comparing trials, the onset of blood lactate accumulation occurred at a significantly lower VO_2_ for the hypohydrated trial. Similar observations were observed by Kenefick and colleagues [45], where collegiate endurance athletes, decreasing their body mass by 3.9% via moderate-intensity exercise, observed the lactate threshold to occur significantly earlier during a treadmill-based graded exercise test. Taken together, the lactate threshold may be affected by large rapid decreases in body mass; however, refeeding and rehydrating might mitigate any negative consequences of RWL on the lactate threshold. Furthermore, it is possible that the decrease in body mass demonstrated by Papadopoulos and colleagues [43] was not of sufficient magnitude to have a substantial impact on the lactate threshold. To recall, combat athletes have been observed to decrease their body mass by more than 10% at times. As such, the roughly 2.5% decrease demonstrated by Papadopoulas and colleagues [43] may not be reflective of typical weight changes in combat sports. In addition, the differences in weight reduction strategies (dietary restriction vs. exercise vs. sauna) may have influenced the observed results for lactate. Previous research showed that individuals who decreased their body mass by 5% through the use of saunas or diuretics within a 24-h period demonstrated greater lactate concentrations during maximal-intensity exercise, compared to a 5% reduction by active exercise over 48 h [46]. Collectively, the lactate threshold as an indicator of metabolic function may be influenced by RWL that induces hypohydration; however, implementing a recovery period may negate the negative impacts. 

Insulin responses to RWL in combat athletes have also been observed in recreationally active males undergoing a 4-day period of LEA, resulting in a weight loss of 2 kg. Participants completed two separate 4-day conditions of low energy availability (~16 kcal·kg^−1^·FFM·day^−1^), which were achieved either through dietary restrictions or exercise. Following the 4-day LEA period, participants demonstrated a significant decrease in resting fasted insulin (−34 to −38%) and increased serum free fatty acids (+70–112%) [38]. 

#### 3.1.3. Future Directions

**Figure 1:**
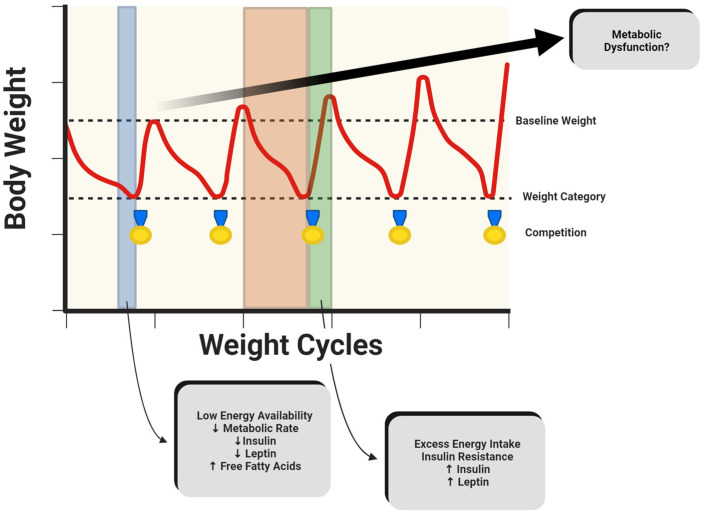
Hypothetical model of metabolic perturbations occurring over time that may potentially increase risk of metabolic dysfunction in combat athletes. The blue area indicates a period of rapid weight loss, while the orange and green areas represent competition preparation and recovery, respectively. Created with BioRender.com (15 January 2024).

For combat athletes to achieve substantial decreases in body mass in a short period of time, a state of LEA is often observed, which may be associated with acute decreases in insulin and leptin and increases in free fatty acids (Figure 1). Collectively, these alterations in metabolic rate and biomarkers, such as insulin and leptin, represent large disruptions in energy and substrate homeostasis [38] and are associated with impaired physiological function or relative energy deficiency in sports [47]. While substrate utilization has been observed between varying levels of fitness [42], minimal research has evaluated substrate utilization specifically in combat athletes. With the training intensity, type, and duration potentially influencing substrate utilization, future research may aim to assess oxidation rates before and after a RWL intervention in combat athletes, using carbohydrate and fat oxidation rates to calculate metabolic flexibility. This may be done utilizing the methodologies outline previously by San-Millán and colleagues [42]. Additionally, with various methods of RWL, research should differentiate the impacts of separate interventions used to decrease body mass, such as passive dehydration through sauna use, low-intensity exercise, and fluid or caloric restrictions. Lastly, with recovery periods in combat sports ranging from minutes to days, future investigations may distinguish the effects of the recovery duration on substrate utilization.

### 3.2. Medium Term

For combat athletes, medium-term perturbations can be defined as events that occur over weeks to months and generally refer to a period of time that describes a ‘fight camp’ or a competition season. Fight camps typically span multiple weeks and may consist of 5–12 training sessions per week, with 2–4 of these sessions dedicated to resistance training [48]. During this time period, combat athletes also typically undergo gradual weight loss or weight cycling. In a sample of 431 college and high school wrestlers, it was identified that 41% of the college athletes had weight fluctuations of 5.0 to 9.1 kg each week over the course of a season [49]. In addition, previous studies have observed that combat sports athletes decrease their body mass by as much as 10–15% in the weeks leading up to a competition [50,51]. Moreover, mixed martial arts athletes have been observed to demonstrate rapid weight regain of approximately 8% in the 24–36 h following weigh-ins and prior to competitions [52], with lighter athletes demonstrating greater weight regain in the week following RWL [53].

#### 3.2.1. Combat Athletes

A previous review by Franchini [54] highlights the large proportion (approximately 50–90%) of energy contribution during simulated combat sports matches stemming from the oxidative system. This range of oxidative system contribution differs between combat sports and depends on the duration of the competition. For example, simulated karate matches have been observed to demonstrate oxidative contributions of 78% [55], while simulated boxing matches demonstrated a contribution of 86% [56]. Additionally, when comparing oxidative contributions in judo matches, a 1-min match resulted in a contribution of approximately 50%, whereas a 5-min match resulted in a contribution of 81% [57]. This information potentially demonstrates parallels, as well as a large difference, between the energy utilization of endurance athletes and combat sports athletes. However, the addition of high-intensity intervals in combat sports, as demonstrated by the work:rest ratios provided previously [40,41], and the subsequent effect on metabolic flexibility have yet to be examined. In addition, another consideration for combat sports athletes is the potential for prolonged periods of reduced energy intake and weight loss while maintaining or potentially even increasing physical activity levels. Kirk and colleagues [51] observed minimal variance in training load during an 8-week fight camp, with no difference observed between competitors losing up to 15% body mass and non-competitors engaging in weight maintenance. 

The effects of prolonged periods of reduced energy intake have been observed in case studies on combat athletes [34,58]. Two athletes, a male international taekwondo competitor and a male professional MMA competitor, decreased their body mass by 13.5% and 18.1%, respectively, over an 8-week fight camp [34,58]. Following the dramatic decrease in body mass, both athletes observed large decreases in testosterone below clinical reference ranges (<5 mmol·L^−1^), as well as a decrease in resting metabolic rate (Kasper [58]: −331 kcal·day^−1^, Langan-Evans [34]: −257 kcal·day^−1^). In general, reduced energy intake and maintained exercise levels could have a negative impact on metabolic function in combat sports athletes when followed by a period of excessive caloric intake after competition. This was observed in the case study of the taekwondo athlete [34], where, during the recovery period after a weigh-in, excessive energy intake (53–98 kcal·kg·FFM·day^−1^) resulted in hyperinsulinemia at the day (142 pmol·L^−1^) and at the week (77 pmol·L^−1^) after the weigh-in, potentially indicative of insulin resistance. A decrease in energy expenditure followed by a hypercaloric diet once an athlete ‘makes weight’ may ultimately result in an increase in body mass beyond the original baseline, making subsequent weight reduction phases more aggressive [59]. In the two weeks following a competition, a case study of a professional male mixed martial artist observed an increase in body mass of 4.2% when compared to baseline body composition assessments taken before the initiation of a fight camp [58]. 

#### 3.2.2. Non-Combat Athlete Comparisons

Regular physical activity has been observed to have numerous beneficial adaptations for sedentary, overweight, and obese individuals, including increased mitochondrial volume, density, function, and content [13]. As such, increased physical activity and exercise are generally associated with improved metabolic flexibility. In a study comparing physically active individuals (4–6 h·week^−1^) to running athletes (16–20 h·week^−1^), the athletes demonstrated greater metabolic flexibility, measured by carbohydrate and fat oxidation, and more efficient anaerobic contributions, during a graded exercise test consisting of incremental speed increases of 0.5 m·s^−1^ from 1.5 to 4.5 m·s^−1^ [60]. Physically active individuals demonstrated significantly lower fat oxidation at speeds of 3.0 and 3.5 m·s^−1^ and significantly greater carbohydrate oxidation at 2.5, 3.0, and 3.5 m·s^−1^. Moreover, when assessing energy system contributions during the graded exercise test, physically active individuals demonstrated significantly higher glycogen system contributions from 1.5 to 3.5 m·s^−1^ when compared to the running athletes. However, this relationship may be influenced by the exercise intensity and training frequency. Additionally, although previous research has shown females to demonstrate greater rates of fat oxidation during exercise when compared to males [61], limited research has investigated metabolic flexibility in females of varying fitness levels or made sex-based comparisons. However, in a study conducted by Olenick and colleagues [62], 25 recreationally active females completed VO_2peak_ testing and an interval exercise bout consisting of four 4-min high-intensity intervals at an intensity at the midpoint between the gas exchange threshold and VO_2peak_ and three 3-min intervals of passive recovery on a cycle ergometer. The participant group was split into groups categorized as high-fitness (VO_2peak_ = 43.7 ± 4.7 mL·kg^−1^·min^−1^) and low-fitness (VO_2peak_ = 33.6 ± 3.9 mL·kg^−1^·min^−1^) based on the achieved VO_2peak_ values. Similar to the previously described observations, females classified as high-fitness demonstrated greater fat oxidation than those classified as low-fitness [62].

Although not specific to combat athletes or exercise, Erdmann and colleagues [63] conducted a study where a group of 10 healthy males with a BMI of <25 increased their BMI by 2 kg/m^2^ over 139 ± 26 days by increasing their daily intake by 300–500 kcal while maintaining their normal levels of physical activity. Following the increase in body mass, the baseline insulin concentrations, insulin secretion, and insulin resistance were observed to increase, while insulin clearance decreased. This study demonstrates that although insulin resistance is typically associated with overweight and obese individuals, insulin resistance may occur in healthy normal-weight individuals when excess calories are consumed and the body mass increases, even when the BMI remains under 25 kg/m^2^.

Similar to the observation in combat athletes, the simultaneous impact of training and chronic LEA in male cyclists was associated with significantly lower testosterone levels when compared to individuals with sufficient energy availability [64]. Additionally, a previous narrative review conducted by Alwan and colleagues [65] outlined the physiological effects of weight management practices and LEA in female physique athletes, including decreases in testosterone, thyroid hormones, and leptin following large decreases in body mass prior to competition [66]. This may ultimately serve as a resource to understand the potential impacts of LEA on female combat athletes. 

#### 3.2.3. Future Directions

Collectively, individuals of normal weight have been observed to demonstrate insulin resistance when undergoing excess caloric intake, regardless of BMI status. This may be of direct concern for combat sports athletes, who typically experience a period of rapid weight gain after weigh-ins and competitions. An additional strategy to make weight for combat athletes is to decrease their body mass over weeks to months. A previous review in overweight and obese individuals [67] has identified that gradual weight loss has fewer negative influences on metabolic function, compared to RWL. Future research may aim to identify differences in metabolic alterations in substrate utilization when comparing RWL with gradual weight loss. Furthermore, with the present manuscript outlining potential negative implications related to excessive caloric intake after competition, metabolic alterations in substrate utilization in the weeks following a competition are of interest. Future investigators may wish to conduct a standardized investigation in combat athletes where the carbohydrate and fat oxidation rates are used to observe changes in metabolic flexibility and biological samples are analyzed to identify changes in markers of metabolic function, before and after a period of rapid weight gain, with comparisons between sex and weight categories. Moreover, post-competition diet interventions such as ‘reverse dieting’ have been deemed popular in populations such as physique athletes, which incrementally increase the caloric intake over an extended period of time to recover the baseline metabolic rate [68]. Distinguishing differences between ‘reverse dieting’ and ad libitum caloric intake and their effects on metabolic function could provide important information on nutritional strategies to optimize overall health in combat sports athletes. 

### 3.3. Long Term

For combat athletes, long-term perturbations can be defined as occurrences that take place over months to years, generally encompassing multiple fight camps, competitive seasons, weight class changes, their athletic career, and life after retirement. Using a questionnaire, Steen and Brownell [49] identified that 35% of their sampled college and high school wrestlers claimed to have lost 0.5–4.5 kg over 100 times in their lifetime. An additional concern is the age at which combat sports athlete may begin these practices. A study conducted by Berkovich and colleagues [69] in adolescent judo athletes identified that the average age at which athletes began practicing RWL was 12.5 ± 2.2 years old. Furthermore, even during a critical stage of physical development, 16% of the participants worked to not gain weight for a period of at least 2 years to remain in the same weight category, and 76.5% of the participants used RWL interventions to do so. Although little research has documented the career lengths of different combat sports athletes, a study with a small sample of identified judo athletes (*n* = 10), described as elite, demonstrated a mean planned retirement age of 30 years old [70]. Taken together, this demonstrates the extensive period of time in which combat sports athletes may be employing weight cycling interventions. 

To date, most evaluations of the long-term effects of weight loss and regain on metabolic function and health status have been conducted in populations defined as overweight or obese, with little research conducted in combat athletes. Multiple long-term beneficial physiological adaptations occur as a result of weight loss in overweight or obese individuals, including increases in mitochondrial efficiency and hormonal alterations to decrease energy expenditure [68]; however, minimal research has addressed the impact of consistent large weight fluctuations over time, specifically in combat sports athletes. Steen and colleagues [71] obtained resting metabolic rates for 27 high-school wrestlers, where athletes were categorized as weight cyclers or non-cyclers (cyclers: *n* = 14, non-cyclers: *n* = 13). Ultimately, weight cyclers (1585.1 kcal^−1^·day^−1^) were observed to have a significantly lower mean resting metabolic rate than non-cyclers (1841.4 kcal^−1^·day^−1^) when matched for weight. However, this appears to be the extent of long-term research on metabolic function in combat athletes.

Although not specific to combat sports athletes, Zhang and colleagues [72] conducted a study with a sample of 644 Japanese men between 40 and 49, looking at weight fluctuations over time (weight at ages 20, 25, 30, and 5 years prior to completion of the survey) and potentially associated with metabolic syndrome. Individuals were subdivided into quartiles of weight fluctuations. Odds ratios for components of metabolic syndrome (hypertension, high fasting glucose, etc.) were provided for each quartile where a linear trend (*p* < 0.015) was observed between the components of metabolic syndrome (high blood pressure, elevated triglycerides, low high-density lipoprotein cholesterol, high fasting glucose, and obesity) and higher weight fluctuations. However, a BMI above or below 25 kg/m^2^ was then used as a confounding factor. A linear trend (*p* = 0.006) was noted between weight fluctuations and metabolic syndrome only for individuals in the BMI ≤ 25 kg/m^2^ group, demonstrating an increased risk of metabolic syndrome with increased weight fluctuations in individuals with a BMI ≤ 25 kg/m^2^. This may be indicative of weight fluctuations over time that demonstrate adverse metabolic effects more predominantly in those at a normal weight. 

Saarni and colleagues [18] observed weight changes in former athletes from non-weight category sports, former athletes from weight category sports, and non-athlete conscript controls identified from the service register of the Finish Defense Forces, over approximately 15 years, with respect to weight at age 20. Individuals who had previously participated in a weight category sport had the highest mean weight gain between their initial measurement and 10-year follow-up. Furthermore, weight category athletes were significantly more likely to be classified as obese (BMI) than athletes from non-weight category sports at all time points. Moreover, when adjusting for current life habits such as post-retirement physical activity, the likelihood of obesity was not affected, influencing the authors to propose weight loss practices during their time as a competitive athlete to modify their weight development post-career. Although outside the scope of this review, post-career weight development may also be related to the psychological aspects of RWL and/or weight cycling, which have been reviewed elsewhere [6]. Additionally, Saarni and colleagues [18] defined previous weight category athletes as weight cyclers due to the common weight reductions in these sports, but there was no information regarding actual weight fluctuations or estimation. As a whole, individuals with a history of weight cycling or weight category athletes may demonstrate a decreased metabolic rate, increased prevalence of metabolic syndrome, and increased risk of obesity later in life.

#### Future Directions

As noted previously, there is little to no research that assesses the negative health implications of weight cycling in sports settings on metabolic function and health longitudinally. Additionally, previous research has noted that unless maintaining recommended physical activity levels, former athletes do not appear to demonstrate any metabolic advantage when compared to non-athletes [21]. As such, former combat athletes who experience metabolic dysfunction as a result of weight cycling without maintaining physical activity levels following retirement may be at a greater risk for increased body mass and factors related to metabolic dysfunction. Future research may aim to combine the previous sections and identify differences in substrate utilization and additional factors of metabolic function in competitive combat athletes during the off-season, a competition training period and a phase of RWL, with continued monitoring after the athlete retires. This may ultimately allow for a comprehensive understanding of how repeated weight cycling throughout a combat sports career may influence metabolic health later in life. Furthermore, during the three separate timeframes outlined in this review, most of the samples were exclusively male participants. As such, it is of interest for each future direction to this point to also emphasize the inclusion of female participants, to better identify and understand any biological sex-based differences in the influence of weight cycling on metabolic flexibility and long-term health.

## 4. Practical Applications

**Figure 2:**
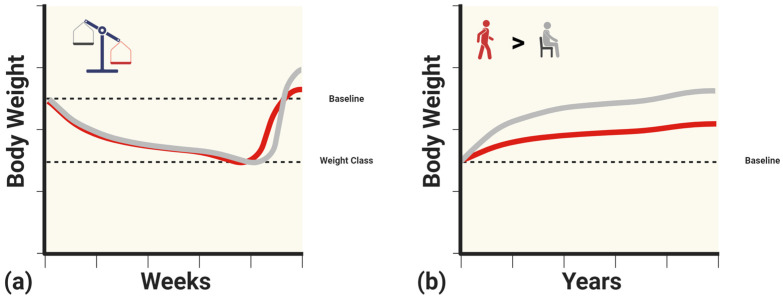
(**a**) Hypothetical illustration of excessive body weight regain beyond the original baseline weight with combat sports athletes in lighter weight categories (gray) compared to those in heavier weight categories (red). (**b**) Hypothetical illustration of continued weight gain following retirement in active (gray) versus sedentary (red) former combat sports athletes. Created with BioRender.com (15 January 2024).

Despite prominent research highlighting ethical concerns, rapid weight loss and weight cycling are common in combat sports. This cycle can lead to physiological changes in the short, medium, and long term, increasing the risk of metabolic dysfunction (Figure 1). Combat athletes undergoing rapid weight loss may experience decreases in metabolic rate, insulin, and leptin. These metabolic adaptations are of greater concern during weight regain after weigh-ins or competition, when excess calories are consumed. As such, it has been observed that combat athletes may exceed their original baseline weight, with lighter athletes experiencing greater weight regain [53] (Figure 2a). This weight gain may be accompanied by signs of insulin resistance. Although not extensively studied, reverse dieting, a strategy commonly used by physique athletes, may serve as a possible intervention to mitigate drastic shifts in metabolism and body composition associated with excessive caloric intake. If weight cycling continues over time, body mass may continue to increase, along with accompanying changes in metabolic function. As such, retired individuals who do not meet the recommended daily physical activity levels may experience even greater increases in body mass and may not be protected from the risk of metabolic syndrome, regardless of their previous athletic status [21] (Figure 2b).

## 5. Conclusions

In the context of combat sports, athletes often engage in rapid body mass reduction before competitions, followed by periods of significant energy consumption and swift weight regain. Notably, during these high-energy intake phases, healthy individuals have shown signs of hyperinsulinemia, which could suggest insulin resistance This is particularly concerning for combat sports athletes, who frequently exhibit extreme caloric consumption post-competition, occasionally leading to a body mass exceeding their initial weight. Several previous investigations have made efforts to address the potential influences of weight cycling on factors of health such as obesity [73] or cardiometabolic diseases [22]; however, the findings appear to be conflicting. Furthermore, while inconclusive, existing research points to potential long-term negative health implications related to weight cycling, such as metabolic syndrome. Since metabolic syndrome is linked to metabolic inflexibility, the acute metabolic changes observed in these athletes might contribute to this syndrome or other metabolic dysfunctions.

Although weight loss in general is associated with improved metabolic flexibility, including enhanced insulin sensitivity, this effect may not be as pronounced in healthy individuals who are neither overweight nor obese. RWL can lead to various metabolic implications, including a possible reduction in lactate threshold during exercise, a decreased metabolic rate, and alterations in insulin and leptin levels. However, it appears that metabolic disturbances are more pronounced during rapid weight gain periods. Proper recovery after RWL might help to mitigate the adverse effects on mitochondrial function. Additionally, maintaining the recommended physical activity levels following retirement may assist in mitigating the potential risks of metabolic syndrome. In summary, while the general population has shown negative metabolic consequences associated with weight cycling, the specific impact on combat sports athletes remains less clear due to limited research. As such, definitive conclusions in this area have yet to be established.

## Data Availability

No new data were created or analyzed in this study. Data sharing is not applicable to this article.
